# “I’ll use it differently now”: using dual-systems theory to explore youth engagement with networked technologies

**DOI:** 10.17269/s41997-020-00347-w

**Published:** 2020-07-08

**Authors:** Valerie Michaelson, Valerie Steeves

**Affiliations:** 1 Department of Health Sciences, Brock University, St. Catharines, ON L2S 3A1 Canada; 2 Department of Criminology, University of Ottawa, Ottawa, ON Canada

**Keywords:** Youth participatory action research, Social media, Networked technologies, Adolescent, Disconnection, Dual-systems theory, Reflexive practice, Recherche-action participative avec des jeunes, médias sociaux, technologies réseau, adolescents, déconnexion, théorie des systèmes duels, pratique réflexive

## Abstract

**Objectives:**

Many young Canadians experience high levels of networked connectivity, which some suggest may negatively impact their health. Adult monitoring has not been shown to be an effective long-term strategy for supporting young people in healthy engagement with tech. In this study, we explore the benefits of empowering young people to set healthy goals and monitor themselves. We engage with Shapka’s (2019) critique of dual-systems theory, and consider the relationship between the neurological and behavioural systems in relation to adolescent internet use.

**Methods:**

Using a youth participatory action research approach, we co-designed a project with six adolescents to explore the ways that their use of networked technologies was affecting their lives by disconnecting and observing how the lack of networked connectivity changed their experiences. The youth used a media diary to track their use of devices both before and after disconnecting.

**Results:**

The main benefit of disconnecting appeared to be having the opportunity to reflect on one’s own use of networked devices. This enabled the participants to reconnect in a more intentional way. Findings support Shapka’s speculation that dual-systems theory, with a focus on regulation, may not be the most useful way of supporting adolescents in developing healthy habits around their wired tech.

**Conclusion:**

Adolescent experiences of networked technologies are complex, yet they are able to navigate this landscape with intelligent strategies. Their self-directed exploration of disconnection helped them to become reflexive practitioners who were able to revisit their use of networked technologies with new insights and self-control.

## Résumé

**Objectifs:**

Les niveaux élevés de connectivité réseau sont courants chez les jeunes Canadiens, ce qui pourrait avoir des effets nuisibles sur leur santé. La surveillance par les adultes n’est pas une stratégie efficace à long terme pour aider les jeunes à établir une relation saine avec la technologie. Nous explorons ici les avantages de donner aux jeunes les moyens de se fixer des objectifs sains et de s’autosurveiller. Nous abordons la critique par Shapka de la théorie des systèmes duels (2019) et considérons la relation entre les systèmes neurologique et comportemental en lien avec l’utilisation d’Internet par les adolescents.

**Méthode:**

À l’aide d’une démarche de recherche-action participative avec des jeunes, nous avons conçu, en collaboration avec six adolescents, un projet pour explorer les effets de l’utilisation des technologies réseau dans leurs vies en les déconnectant et en observant en quoi l’absence de connectivité réseau modifiait leur expérience. Les jeunes ont noté leur utilisation d’appareils en réseau avant et après la déconnexion dans un « journal médias ».

**Résultats:**

Le principal avantage de la déconnexion semblait être la possibilité de réfléchir à sa propre utilisation des appareils en réseau. Les participants ont ainsi pu rétablir cette connexion de façon plus intentionnelle. Nos constatations confirment l’hypothèse de Shapka selon laquelle la théorie des systèmes duels, axée sur la réglementation, n’est peut-être pas le meilleur moyen d’aider les adolescents à acquérir de saines habitudes face à leurs appareils en réseau.

**Conclusion:**

L’expérience des technologies réseau chez les adolescents est complexe, mais avec des stratégies intelligentes, ils réussissent à trouver leur voie. Leur exploration autodirigée de la déconnexion les a aidés à devenir des praticiens réfléchis, capables de retourner à leur utilisation des technologies réseau avec plus de retenue et des vues plus larges.

## Introduction

Many young Canadians experience high levels of connectivity through their use of social media and networked technologies. A 2014 survey reports that access is almost universal (99%); moreover, the majority of children use portable devices, such as smart phones and iPods, to connect to the internet, making it easier for them to go—and stay—online throughout the day (Steeves 2014).

A growing body of literature posits that such high levels of connectivity may have negative impacts on children’s physical, psychological and social health (Wartella et al. 2000; Yao and Zhong 2014; Guan and Subrahmanyam 2009; Wallace 2014). A 2018 report from the US-based Pew Research Centre reported that nearly half of 13- to 17-year-olds are also worried, and think that they spend too much time on their cellphones (Jiang 2018).

Yet, adult interventions such as monitoring usage (Shapka and Law 2013; Shade and Singh 2016) are unlikely to change the amount of time youth spend on their devices (Elsaesser et al. 2017; Erickson et al. 2016). When adults do micromanage young people’s technology use, the results are often a weakened parent/child bond (Shapka and Law 2013) and feelings of guilt and shame (Shapka 2019). Moreover, when adults are overbearing around technology use, young people will often take their networked activities underground. This exacerbates problems around usage, because young people now have even less access to support from adults who might help them if they run into trouble online (Shapka 2019; Midamba and Moreno 2017).

Adult monitoring and goal setting of adolescent activity online has not been shown to be an effective long-term strategy for supporting young people in healthy engagement with wired tech. What if instead of focussing on controlling young people, the focus was on supporting them to identify and set their own healthy goals for their networked activities? Essentially, what if young people were empowered to monitor themselves? Our current study engages with these questions.

## Theoretical framework and methodology

Our study was informed by Shapka’s (2019) insightful critique of dual-systems theory, which has been used to understand people’s use of wired devices and the internet (Soror et al. 2015; Osatuyi and Turel 2018; Turel 2015; Turel and Qahri-Saremi 2016; Turel and Serenko 2012). This theory proposes that behaviour is influenced by two distinct systems (Shapka 2019; Osatuyi and Turel 2018; Soror et al. 2015): the neurological system, which involves reasoned and goal-directed decision making (Shapka 2019; Strack and Deutsch 2004); and the behavioural system, which involves one’s automatic and often unconscious behaviours (Shapka 2019; Strack and Deutsch 2004). While these two systems can work together, much of the research that examines behaviours such as gambling, over-eating and internet addiction assumes that they operate in conflict (Turel and Qahri-Saremi 2016), and that an individual’s excessive or addictive online use relates to poor self-regulation skills. From this perspective, the impulsive behavioural system wins a battle over the more reasoned neurological system’s goals, resulting in excessive screen use.

While this appears to be the case for many adults (Soror et al. 2015; Osatuyi and Turel 2018; Turel 2015; Turel and Qahri-Saremi 2016; Turel and Serenko 2012), Shapka questions whether or not this assumption holds true for adolescents who likely have very different goals regarding their wired technologies than adults, particularly in terms of their social needs. For example, connecting with other teens online may be useful to adolescents who, as part of their neurological development, are seeking to separate from the family and experiment with their own identities through their membership in peer groups (Buckingham 2008; Gross 2004). As Shapka observes, “most adolescents likely do not think of going online or using their mobile device as a bad habit that they need to overcome” (Shapka 2019, p. 106). If there is no problem in the first place, there is no need for regulation; the two systems are simply operating together in a reasonable way. Moreover, what appears to be the case for many adults in terms of how behavioural and neurological systems work against each other may not hold for adolescents, who have very different priorities in terms of how and why they use their social media and tech. Building on Shapka’s critique, we view data generated from this study through the lens of dual-systems theory. We engage with the question as to whether or not these two systems actually are at odds in terms of adolescent internet use, or if in fact something more complex is at play.

Our methodological approach was driven by the assumption that the best people to provide insight into the relationship between young people and networked tech are young people themselves. Thus, we used youth participatory action research (YPAR) methods to co-design a project with young people to evaluate their subjective experiences with both connection and disconnection. YPAR’s goal is to engage individuals who have personal experience of the research topic as co-researchers in order to learn more about issues that are important to the individuals themselves (Ozer 2017). This particular project emerged from our ongoing work with a well-established community of young people who were interested in adolescent health. The youth identified social media and electronic device use as an interesting adolescent health topic for them to explore^1^ and then worked in partnership with the authors to co-design and co-implement a project to learn more about how their connection to networked technologies was impacting their health.

### Demographic information

Six youth (two boys and four girls) between 11 and 15 years of age participated in the study. All six lived in a medium-sized city in Ontario. Although participants were not recruited on the basis of self-identification with regard to race, ethnicity, or gender identity, our participant group included members of racialized minorities and various religious groups.

### Establishing the research partnership

We began by collectively reviewing existing research about young people’s use of networked technologies, looking in particular at the findings of MediaSmarts’ Young Canadians in a Wired World research project.^2^ After discussing the existing state of knowledge with the authors, the teens brainstormed a number of possible research topics. Consensus arose around the goal of better understanding how the high degree of connectivity they were experiencing in their daily lives was affecting their relationships. Working with the authors, they decided to operationalize the impact through the concept of connectedness, and posed the following research question: How do networked technologies impact our sense of connection with ourselves, other people and nature?

### Project design

The group mapped their networked connectivity by working in small groups to generate a list of the devices and apps they use on a regular basis, and a list of the activities they do on those devices and apps. They then proposed that a good way to study the ways that these devices and activities affect their lives was to disconnect from them and see how the lack of networked connectivity changed their experiences of connectedness. Ethical approval for the project was attained from Queen’s University (ROMEO/TRAQ #6017552) and University of Ottawa (File Number: 09-16-25) by January 2017, and from Brock University (File 19-087) in 2019.

The youth tracked their device use before and after disconnecting in a media diary that they co-designed with the authors. The diary included a checklist of 14 distinct categories of how and why they used networked devices. The full list is found in Appendix 1. They kept the diary for one week to map their existing tech use and their sense of connection to self, others and nature. They then took a media fast, intentionally disconnecting from electronic devices for seven consecutive days during the next two-week period, and kept a diary of their experiences of connection.

The youth set the parameters around the experiment, determining what they would and would not do during the fast. Although they wanted to forgo all screen use, they decided that it would be impractical, if not impossible, to keep a full “fast,” because the adults in their lives (parents, teachers, employers and coaches) required them to remain connected. Because of this, they agreed on a “media diet.” While they fasted from most of their social media and other screen use, they continued with things like texting parents to pick them up, doing homework, watching movies with their families, and communicating with part-time employers. The guidelines for their fast/diet are found in Appendix 2.

### Interviews and coding

After the fast, the authors interviewed each youth researcher in order to provide a space for individual reflection on the experience, which we then brought to the larger group. We used open-ended questions to encourage the youth to reflect on their experiences before, during and after the media fast. The interviews were audiotaped and transcribed by a professional transcriber for analysis. All identifying information was removed from the transcripts, and pseudonyms were used to identify participants.

The youth researchers decided not to read the transcripts because they were concerned that they would be able to identify each other. The authors accordingly read all transcripts and used open coding methods to identify emerging themes using a grounded theory approach. The authors then reported back to the group, explaining the various themes they had identified and pulling out quotes to illustrate each theme. We then met as a group to discuss how to interpret the themes. In this way, we worked hard to ensure that interpretive authority over the transcripts remained in the hands of the young people. While the youth had the option of sharing with the group when one of their own quotes was being discussed during our interpretation meeting, the adult interviews made it clear that they would not divulge this information. This entire process was proposed and guided by the youth researchers.

## Results: Intentional disconnection and its impact on tech use

### Self-regulation

When our young co-researchers decided for themselves that they wanted to disconnect from their technologies, they were readily able to do so. Indeed, as Evan said, “It wasn’t nearly as bad as I thought it was going to be.” While initially Kian thought that the media fast would “be a big thing to do… after you’ve tried it, it’s not that bad.” Zoe agreed: “The first day I was kind of wondering about [my Netflix series] and then I sort of forgot.… I thought that if I had seen almost all of it I would be wondering what was going to happen and be stuck in suspense for a week. But it was okay… I thought that since I spend so much time doing that kind of thing that it would really impact me. But I realized that it doesn’t have too much meaning to it and it isn’t a really important part of my day. So when I took it out I was like, whatever I don’t really care.” Evan described feeling “surprised and proud at the same time … Some people were like, oh I bet you can’t go a week without electronics. And I think they are surprised that I did it. It was a proud and a surprising feeling. And it wasn’t that bad except for one day.” For Maya, “the first days were probably like the hardest, because I just find there was a lot of time where I kind of didn’t have anything to do, so I just kind of automatically want to, I dunno, go through Instagram or something… but then I couldn’t…. At first it felt kind of weird but then after that I just got like more used to it. So I stopped really noticing that I wasn’t using it at all.” For these particular adolescents, their self-regulation around this project was impressive, but it was also understandable. After all, this was their project and they had set the parameters themselves. Kian shared about how his friends had questioned his intention to take the media fast seriously. “Some of them said, do they know that you’re not going on? Because they said well if they don’t know why don’t you just go on? I thought, oh, that’s not the point, that’s not the point of the experiment.”

As we worked on this project together, an interesting paradox emerged. The main barrier the youth experienced in keeping a full media fast was not Netflix, social pressure from friends, or video games, but pressures from the adults in their lives around staying connected for the sake of homework, scheduling and communication. As adults ask critical questions about how much time young people spend on screens, it is worth also reflecting on the current reality that adults have created a world that demands nearly constant connection from young people, and this reality makes it very difficult to disconnect. Initially, Zoe had even wanted to cut out all tech—even the parts that the group agreed they would continue to do—for a week and “see how it felt.” She reflected: “But then I realized that I couldn’t really do it. And not so much for personal reasons. It was more really practical reasons. And I realized that I depend on it a lot more for stuff like school. For each class we used it for so many important things and if I cut it out for one day I would have so much to catch up on.” Young people may be caught in a Catch-22 kind of situation in which parents, teachers and other adults in their lives are simultaneously concerned about the amount of time they are using their wired technologies, and imposing top-down regulations and strategies to control their technology use, while also insisting on a high level of constant connection for their own agendas. In relation to Shapka’s (2019) insights about regulation, it is noteworthy that in the context of this study, adults appeared to be regulating not the connectivity, but the limits on how far the young people’s disconnection experiment could go.

### Self-awareness

The participants reported that the act of intentionally disconnecting gave them ways to become more reflexive about their use of networked devices. “I don’t think we realize how much we do use technology,” Naomi reflected. “We just think that it is… it is just part of our routine and so we don’t realize what we are doing. But then when I wasn’t using it for a week I realized that I do use it a lot even though I didn’t think that I did. The diet made me more aware of how much I wanted to use it and to talk to my friends and send a bunch of stuff. So that was cool to think about. And then I realized how much I do use technology every day.” Maya, too, talked about how she did not think she used technology very much, but during the fast, she was surprised by how much time she had left when she “wasn’t allowed to use social media or anything.”

Along with this emergent self-awareness, all of these youth expressed that their tech wasn’t as important to them as they thought it was going to be. Zoe knew this “because when [she] stopped doing it [her] life did not dramatically change.” She would not choose to “keep up the fast forever” but it “made [her] realize that it wasn’t as important as [she] thought it was.” Kian agreed: Technology is “not as big of a part as you think it is. Like, you never really notice that until you just take it away.” These youth recognized that their tech use was perhaps more habitual than they had realized, aligning with the theory that habits, regulated by the behaviour system, impact use of social media and other networked tech. However, whether or not this is in competition with the neurological system, which enables one to self-regulate, is another question. What we observed as we worked with these young people, and about which Shapka (2019) has speculated, is that once they had changed their goals, they were readily able to change their habits to align with their new goals.

Through their intentional disconnection and reflection, these youth were quick to recognize some of the benefits of disconnecting, and emphasized how they found that during the media fast, they had increased time to do homework, sleep, and just sit and think about nothing in particular so that their thoughts had time and space to take shape without distraction. Maya realized that during the fast, she had a lot of extra time too because she “wasn’t wasting it” or “just using it up... on a device.” Olivia found it was easier for her to fall asleep without the distraction of texting, and watching movies late at night. Zoe experienced reduced stress: “I think that it is really mentally exhausting and it takes a lot of effort to keep track of and do all of those things. It is like a whole new aspect to your life that you have to take care of.” Zoe was a bit happier during the fast: “I think I was less stressed when I wasn’t around it. But I don’t think that it made me really angry or mad having it. It was just a lot of added pressure onto your life.” Kian said, “it felt good not to worry about social media.” Maya explained that disconnecting for a week helped her see that sometimes she uses social media to just fill in time, “and spending too much time going through things like Instagram or Pinterest doesn’t make you feel any better.” Evan enjoyed seeing his friends face-to-face more: “It is just better when you are next to each other and not just texting … There is no emotion or tone in texting.” Evan also told us that one day during the fast, he “just sat on bed and was thinking. And was bored.” Eventually, just doing nothing “made him realize things that [he] had not realized before.” He reported that taking this space to think made him more open-minded. As he reflected on this experience, he said: “If there is something that you absolutely hate but you think about it more...I mean maybe you just don’t like this person. But you think about it and what they are going through and why are they acting like such a jerk. So stuff like that. It just opens your eyes and makes you more open-minded which is always better.” One could readily argue that these insights about the benefits of disconnecting are in keeping with displacement theory, which posits that time spent with electronic screens can displace time spent engaging in other activities (Neuman 1988), including sleep (Exelmans and Van den Bulck 2017; Przybylski 2019) and going outside (Subrahmanyam and Šmahel 2011; Gray et al. 2015), and result in a negative impact on health (Houghton et al. 2018). They also reinforce Shapka’s (2019) speculation that for adolescents and their use of networked technologies and social media, the behavioural and neurological systems may not be working in conflict at all. What we saw in our study was that for these young people, the act of making their own observations, in the contexts of their own lives and based on goals they had set independently, provided a space to identify and recognize what had been displaced for themselves, and on their own terms. Essentially, their own self-designed experiment of disconnection enabled them then to adjust their online activity to match their revised priorities and goals.

### Re-connecting as reflexive practitioners

Based on these research findings, the main benefit of disconnecting appeared to be having the opportunity to reflect on one’s own use of networked devices. This in turn enabled the participants to reconnect in a more thoughtful and intentional way. In effect, the project equipped the youth to engage in reflexive practice (Dewey 1910; Nadin and Cassell 2006) about their own use of technology. Reflexive practice is developed through a process by which “individuals (or groups) critically question their experiences to develop new understandings and knowledge that are ultimately reinvested to transform their actions” (Bisset et al. 2015, p. 167–168). Although the data did not directly address how race, gender, sexuality and ethnicity play out in online teen culture, in the end, the youth, who were situated in a variety of social locations, felt that disconnecting gave them a way to regain some sense of control, and to decide for themselves how to use networked technologies in ways that best support their own unique needs. This raises interesting questions about how to balance the desire to support teens as agents in their own right with the need to hold platforms to account for the ways that racism, misogyny and other discriminatory factors shape young people’s experiences online (Baily and Steeves 2015). This also points to the need for further research that can more fully interrogate whether or not the opportunity to disconnect is equally available to all children, independent of their social location.

Despite the benefits to disconnecting, all the youth told us they would still rather have their games, shows and social media. They used tech for important reasons, such as keeping organized, sharing jokes with friends, and staying in contact with parents. Even though they all agreed that face-to-face is better, Olivia noted that when you aren’t with your family, things like FaceTime are appreciated because “then you can see… what they are doing.” Naomi talked about being with her friends at school at lunchtime: “We were on Snapchat and Instagram and we always like to take bad photos of each other but it is hilarious and we are fine with it. And so we will put them on our Story, which is only our friends who can see it. And it will be like, happy birthday. It is funny. So we had a lot of fun taking photos and videos and doing crazy stuff. And basically videos and doing fun stuff.” Evan said: “I don’t absolutely need this to survive. But I would still rather have it.” But there are also times, he told us, that you just want to stay home or be by yourself. For example, a day before the fast, one of his friends wanted him to go to the park. “And I felt like I didn’t want to get up and walk over. So I just said that I couldn’t. Sometimes it feels too inconvenient to get up and put on my shoes and jacket and walk over.” On those days, what he really wanted to do was just play video games. “There are some days where I probably shouldn’t be doing it but I choose video games over hanging out with friends sometimes. Sometimes I am just not in the mood or in the mental state to hang out with people.”

Zoe thinks that cautions around social media use may be over-rated. She described how “People talk about how if you spend too much time on social media then you get really jealous or angry for the rest of the day. I haven’t found that at all.” Evan reflected on criticisms that technology affects how you socialize with people. “If you use technology way too much,” he said, “then that is bad and you will have no social skills. I feel that it is important to have some, unplug the screen time and go outside.” However, his views were more nuanced, and he recognized another dimension: “But I think that technology is also really important. It can be social in the sense that you can meet people through technology and you can socialize and learn about what is going on. Also it makes people open-minded. Because if you hear about what is going on around the world then that is interesting.”

After the fast, the whole group reported that they were using technology differently. Zoe was glad to have her phone back, but she wasn’t spending as much time on Snapchat, or scrolling through Instagram, and she was only going to Snapchat with her close friends from here on. “I think I’m toning it down a bit,” she told us, “I haven’t been caring as much.” Maya, too, was spending less time on Instagram, but also valuing the time she does use tech more, because, “it’s like felt more special right after the fast.” The fast gave Naomi new awareness: “Now that I realize how much I use it and so I think now I am more aware and I will go outside more. And so when I am bored I will be like, okay I should go outside. And then I will go outside and actually have fun.” Olivia found that she checks her phone a lot less often: “Before I would check my phone a few times every hour but now I will go more than an hour without looking at my phone. I feel like over the media fast I didn’t do it so now it is not as much of a habit to check my phone.”

Kian issued these words of caution about networked technologies: “If you’re really into it, it’ll suck you in… Some people won’t talk to other people face to face, they won’t spend time with the family, they won’t go outside… And they might never do stuff that they think is good, or fun, because they’re sucked into it. Like I don’t feel sucked in, really. But, like, a lot of my friends, they just... I feel like they want to be talking, talking to everybody. And they want to, yeah... they just feel the need, that they need to... do this. That it’s part of your life, but it doesn’t have to be … “[Technology is] not as big of a part as you think it is. Like, you never really notice that until you just take it away. … Because you can live without it and there’s nothing that’s stopping from doing that, except you... feeling the need to talk to people and go online and feel good about it.”

Overall, while dual-systems theory may be a useful tool for understanding social media and wired technology use of adults, a degree of caution, and more research, is needed before concluding that it works in the same way for adolescents. Shapka cites Soror et al. (2015) to suggest that a habit is only “bad” when it conflicts with an established goal. When our participants were setting their own goals, they were readily able to self-regulate—or exert self-control over—their tech use in a way that worked well with their lives. Indeed, they were readily able to engage their neurological and behavioural systems in a complementary way. Throughout this project, each participant drew on their own capacity for self-regulation as an important resource. This in turn supported them as they became reflexive practitioners who were able to interrogate and then transform their own practice.

We put forward these data and reflections on dual-systems theory with a degree of caution, recognizing that our small sample size of extremely engaged and articulate young people is a strong factor in our findings. The ability to navigate self-regulation around social media and internet use may be dependent on the individual child. Our findings contribute a strong impetus for testing whether or not young people’s self-regulation around internet and social media use increases when they are the ones in charge of setting their own goals, and suggest that intentional, self-directed disconnection for a limited time helps young people to emerge as more reflexive practitioners who are in control of their own goals and tech use. This paper contributes to an emergent dialogue around the ways that young people are using social media networked technologies by going beyond the current focus on self-regulation to suggest that an important way forward in helping young people to develop healthy online habits may be to focus instead on helping them to become reflexive practitioners who have opportunities to intentionally set their own goals. Health promotion studies of social media often relate to its utility for health promotion campaigns; another contribution this study makes is to enlarge this focus to consider how one’s engagement with new and emergent online contexts shapes health behaviours and outcomes in complex and sometimes unpredicted ways.

## Conclusions

In this study, we engaged with dual-systems theory through a PAR study to understand young people’s engagement with social media and wired technologies. A strength of this project was that it was designed by youth about a question they had chosen themselves. From start to finish, the project was driven by their curiosity, energy, insights and leadership. Because they were empowered as co-researchers, traditional power imbalances in academic research were reset, and they were able to gain understanding about their own lives. In terms of limitations, the small group size means that our study was not generalizable to a large population.

The study supports Shapka’s speculation that dual-systems theory, with a focus on teaching self-regulation, may not be the most useful way of supporting adolescents in developing healthy habits around networked technologies because young people’s experiences are so complex and so unlike the experiences of adults. We argue instead that empowering young people to reflect critically on their own social media use, and to set and monitor their own goals, may be more effective in the long term. Our findings suggest that when young people maintain control over their own lived technological worlds, they have great capacity to use their devices to help them negotiate a complex landscape with intelligent strategies and astute self-reflection. Their self-directed exploration of disconnection helped them to become reflexive practitioners who were able to revisit their use of networked technologies with new insights and self-control.

## Appendix 2. Media diet rules

Things not included in the diet:

- Emails

- Any school-related use

- Emergencies

- Planning

- Netflix on TV or shared computer screen with others (e.g. family)

- Music on radio, iPod without earbuds (e.g. dock)

- Games with other people in the same room

- Taking photos

Things they will not be doing during the media diet:

- Snapchat

- Instagram

- Pinterest

- Netflix on a personal device on their own (e.g. watching Netflix on a laptop by yourself in your bedroom)

- Music on iPod using earbuds (except for school or work)

- Games on a device played by yourself

- Posting photos

- Looking at/through photos

## Footnotes

1 This is consistent with the findings of a 2014 survey of 5436 Canadian students aged 11–17 in which 35% of survey respondents reported being “worried” that they spent “too much time online.” The percentage was surprisingly consistent across grades and even more pronounced for girls (40% compared with 31% of boys) (Steeves 2014, p. 44).

2 This project has been collecting data on youth and technology since 2000 (see http://mediasmarts.ca/research-policy).

## References

[CR1] Baily, J., & Steeves, V. (2015). eGirls, eCitizens. Ottawa: University of Ottawa Press.

[CR2] Bisset S, Tremblay MC, Wright MT, Poland B, Frohlich KL (2015). Can reflexivity be learned? An experience with tobacco control practitioners in Canada. Health Promotion International.

[CR3] Buckingham D (2008). Youth, identity, and digital media.

[CR4] Dewey, J. (1910). Natural resources in the training of thought. *How We Think*, 29–44.

[CR5] Elsaesser C, Russell B, Ohannessian CM, Patton D (2017). Parenting in a digital age: a review of parents’ role in preventing adolescent cyberbullying. Aggression and Violent Behavior.

[CR6] Erickson LB, Wisniewski P, Xu H, Carroll JM, Rosson MB, Perkins DF (2016). The boundaries between: parental involvement in a teen’s online world. Journal of the Association for Information Science and Technology.

[CR7] Exelmans L, Van den Bulck J (2017). Bedtime, shuteye time and electronic media: sleep displacement is a two-step process. Journal of Sleep Research.

[CR8] Gray C, Gibbons R, Larouche R, Sandseter EB, Bienenstock A, Brussoni M, Chabot G, Herrington S, Janssen I, Pickett W, Power M, Stranger N, Sampson M, Tremblay MS (2015). What is the relationship between outdoor time and physical activity, sedentary behaviour, and physical fitness in children? A systematic review. International Journal of Environmental Research and Public Health.

[CR9] Gross EF (2004). Adolescent internet use: what we expect, what teens report. Journal of Applied Developmental Psychology.

[CR10] Guan S-SA, Subrahmanyam K (2009). Youth internet use: risks and opportunities. Current Opinion in Psychiatry.

[CR11] Houghton S, Lawrence D, Hunter SC, Rosenberg M, Zadow C, Wood L, Shilton T (2018). Reciprocal relationships between trajectories of depressive symptoms and screen media use during adolescence. Journal of Youth and Adolescence.

[CR12] Jiang, J. (2018). How teens and parents navigate screen time and device distractions. *Pew Research Center*, August 22, 2018.

[CR13] Midamba N, Moreno M (2017). Differences in parent and adolescent views on cyberbullying. The Journal of Adolescent Health.

[CR14] Nadin S, Cassell C (2006). The use of a research diary as a tool for reflexive practice: some reflections from management research. Qualitative Research in Accounting & Management.

[CR15] Neuman SB (1988). The displacement effect: assessing the relation between television viewing and reading performance. Reading Research Quarterly.

[CR16] Osatuyi B, Turel O (2018). Tug of war between social self-regulation and habit: explaining the experience of momentary social media addiction symptoms. Computers in Human Behavior.

[CR17] Ozer EJ (2017). Youth-led participatory action research: overview and potential for enhancing adolescent development. Child Development Perspectives.

[CR18] Przybylski AK (2019). Digital screen time and pediatric sleep: evidence from a preregistered cohort study. The Journal of Pediatrics.

[CR19] Shade, L. R. & Singh, R. (2016). “Honestly, We’re Not Spying on Kids”: school surveillance of young people’s social media. *Social Media+ Society 2*(4), 2056305116680005.

[CR20] Shapka JD (2019). Adolescent technology engagement: it is more complicated than a lack of self-control. Human Behavior and Emerging Technologies.

[CR21] Shapka JD, Law DM (2013). Does one size fit all? Ethnic differences in parenting behaviors and motivations for adolescent engagement in cyberbullying. Journal of Youth and Adolescence.

[CR22] Soror AA, Hammer BI, Steelman ZR, Davis FD, Limayem MM (2015). Good habits gone bad: explaining negative consequences associated with the use of mobile phones from a dual-systems perspective. Information Systems Journal.

[CR23] Steeves, V. (2014). Young Canadians in a Wired World, Phase III: Life Online. Ottawa: MediaSmarts.

[CR24] Strack F, Deutsch R (2004). Reflective and impulsive determinants of social behavior. Personality and Social Psychology Review.

[CR25] Subrahmanyam K, Šmahel D (2011). Internet use and well-being: physical and psychological effects. Digital Youth.

[CR26] Turel O (2015). Quitting the use of a habituated hedonic information system: a theoretical model and empirical examination of Facebook users. European Journal of Information Systems.

[CR27] Turel O, Qahri-Saremi H (2016). Problematic use of social networking sites: antecedents and consequence from a dual-system theory perspective. Journal of Management Information Systems.

[CR28] Turel O, Serenko A (2012). The benefits and dangers of enjoyment with social networking websites. European Journal of Information Systems.

[CR29] Wallace P (2014). Internet addiction disorder and youth. EMBO Reports.

[CR30] Wartella E, O’Keefe B, Scantlin R (2000). Children and interactive media: compendium of current research and directions for the future.

[CR31] Yao MZ, Zhong Z-J (2014). Loneliness, social contacts and Internet addiction: a cross-lagged panel study. Computers in Human Behavior.

